# Unveiling the role of risk factors and predictive models in acute type-a aortic dissection surgery: OI downregulation and its association with immune disorders

**DOI:** 10.7150/ijms.104622

**Published:** 2025-01-13

**Authors:** Fuyan Ding, Jianyang Liu, Hong Wang, Ying Tan, Zhidong Zhang, Gang Qiao, Taibing Fan

**Affiliations:** 1 Department of Vascular Diseases Intensive Care Unit, People's Hospital of Zhengzhou University, Zhengzhou University Central China Fuwai Hospital, Zhengzhou 450000, China.; 2 Department of Vascular Surgery, People's Hospital of Zhengzhou University, Zhengzhou University Central China Fuwai Hospital, Zhengzhou 450000, China.; 3 Department of Children's Heart Center, People's Hospital of Zhengzhou University, Zhengzhou University Central China Fuwai Hospital, Zhengzhou 450000, China.

**Keywords:** Acute type A aortic dissection, Acute lung injury, Risk factors, Clinical factors, Predictive efficacy

## Abstract

**Background and Objective:** Acute type A aortic dissection (ATAAD) represents a critical and life-threatening condition requiring urgent surgical intervention, which is often life-saving. However, postoperative acute lung injury (ALI) has emerged as a prominent complication that significantly impacts patient outcomes and prognosis. This study aims to systematically analyze the risk factors associated with the development of severe ALI following ATAAD surgery, providing insights to improve postoperative management strategies.

**Methods:** A retrospective analysis was conducted using a comprehensive database comprising 483 patients diagnosed with ATAAD. Patients were stratified into two groups based on the severity of postoperative ALI: severe ALI group (n = 182) and non-severe ALI group (n = 301). Clinical data were systematically collected and compared between the two cohorts. Binary logistic regression analysis was employed to identify independent predictors of severe ALI following ATAAD surgery. The diagnostic accuracy of these risk factors was assessed using receiver operating characteristic (ROC) curve analysis, with the area under the curve (AUC) serving as the metric for prognostic performance.

**Results:** The severe ALI group exhibited a higher prevalence of preoperative oxygenation index (OI) ≤ 200 mmHg, smoking history, and coronary artery disease compared to the non-severe ALI group (P < 0.001, P = 0.032, and P = 0.039, respectively), while the prevalence of Marfan syndrome was lower (P = 0.033). Moreover, significant differences were observed in several clinical and intraoperative parameters, including body mass index (BMI), C-reactive protein (CRP), procalcitonin (PCT), D-dimer, white blood cell count (WBC), aortic cross-clamp time, moderate hypothermic circulatory arrest (MHCA) time, cardiopulmonary bypass (CPB) duration, and ICU length of stay (all P < 0.05). Multivariate logistic regression identified preoperative OI [P = 0.008, OR (95% CI): 0.002 (0.000-0.183)], BMI [P = 0.037, OR (95% CI): 1.569 (1.027-2.397)], CRP [P = 0.022, OR (95% CI): 1.292 (1.037-1.609)], D-dimer [P < 0.001, OR (95% CI): 3.841 (1.820-8.108)], MHCA time [P = 0.001, OR (95% CI): 3.306 (1.670-6.544)], and CPB duration [P = 0.017, OR (95% CI): 1.117 (1.020-1.223)] as independent predictors of severe ALI. ROC curve analysis revealed the diagnostic performance of preoperative OI, BMI, CRP, D-dimer, MHCA time, and CPB duration, with AUC values of 0.715, 0.844, 0.871, 0.955, 0.944, and 0.833, respectively (all P < 0.001).

**Conclusion:** Preoperative oxygenation index, BMI, CRP, D-dimer levels, MHCA time, and CPB duration are independent risk factors for the development of severe ALI following ATAAD surgery. These findings underscore the importance of preoperative risk assessment and perioperative optimization to mitigate the risk of severe ALI and improve patient outcomes.

## 1. Introduction

Aortic dissection (AD) is characterized by a disruption in the innermost layer of the aorta, resulting in blood entering the space between the intima and media to form a false lumen. The pressure-driven blood flow within the false lumen can extend the dissection along the longitudinal axis in an anterograde or retrograde direction, potentially causing rupture of the aorta. This process often establishes communication between the true and false lumens through one or more breaches^1^, ^2^. Based on the extent of aortic involvement, AD is classified into two categories: Stanford type A and Stanford type B. Stanford type A, involving the ascending aorta, accounts for approximately 60-70% of all cases 3, 4.

Acute type A aortic dissection (ATAAD) is one of the most severe and life-threatening cardiovascular emergencies, necessitating prompt surgical intervention to prevent fatal complications such as aortic rupture or multi-organ ischemia. The surgical repair of ATAAD, while often life-saving, remains a formidable challenge due to its inherent complexity^5^, ^6^, prolonged cardiopulmonary bypass (CPB) times, and the need for deep hypothermic circulatory arrest (DHCA) to create a bloodless surgical field^7^, ^8^. Despite significant advancements in surgical techniques and perioperative care, the postoperative outcomes for ATAAD remain suboptimal^9^-^11^, with acute lung injury (ALI) emerging as a frequent and serious complication 12-14.

ALI is a critical form of acute respiratory failure characterized by widespread pulmonary inflammation, increased vascular permeability, and impaired gas exchange, often resulting in intractable hypoxemia 15, 16. In the context of ATAAD surgery, ALI can be triggered by several factors, including the systemic inflammatory response associated with CPB, ischemia-reperfusion injury during DHCA, and hemodynamic fluctuations inherent to the procedure. These pathophysiological insults collectively compromise the integrity of the alveolar-capillary membrane, leading to pulmonary edema and respiratory dysfunction. Reports indicate that ALI occurs in 30-50% of ATAAD surgical cases and is associated with prolonged ICU stays, higher morbidity, and increased mortality rates.

To date, limited research has systematically examined the risk factors contributing to the development of ALI in ATAAD patients. Preoperative oxygenation index (OI), body mass index (BMI), and biomarkers such as C-reactive protein (CRP) and D-dimer have been implicated as potential predictors in other forms of respiratory failure. However, their specific relevance in the context of ATAAD remains underexplored. Furthermore, intraoperative parameters, including CPB and DHCA durations, are known to exacerbate systemic inflammation and ischemia-reperfusion injury, potentially heightening the risk of ALI.

Understanding the interplay of these factors is essential for improving risk stratification and perioperative management. Early identification of high-risk patients could enable targeted interventions, such as optimizing preoperative lung function, mitigating inflammation, and refining intraoperative strategies, thereby reducing the incidence and severity of ALI. Moreover, a comprehensive evaluation of these risk factors could guide the development of predictive models to support clinical decision-making and improve outcomes.

This study aims to bridge these knowledge gaps by systematically analyzing clinical, biochemical, and intraoperative variables associated with ALI in ATAAD surgery. Using a robust retrospective dataset and multivariate statistical modeling, this research seeks to identify independent risk factors and assess their diagnostic value through receiver operating characteristic (ROC) curve analysis. These findings will not only contribute to the understanding of ALI pathogenesis but also offer practical insights for improving perioperative care and prognosis in ATAAD patients.

## 2. Methods

### 2.1 Inclusion criteria

The inclusion criteria for this study required patients to have a confirmed diagnosis of acute type A aortic dissection (ATAAD) using advanced imaging techniques such as computed tomographic angiography (CTA), magnetic resonance angiography (MRA), or transesophageal echocardiography (TEE), with hallmark imaging features including intimal tears and false lumen formation^17^. Furthermore, all cases adhered to the established diagnostic criteria for ATAAD and presented within 14 days of symptom onset, aligning with the recognized temporal definition of acute dissection. Eligible patients underwent surgical intervention for ATAAD, and to ensure comprehensive analysis, complete preoperative clinical records and postoperative monitoring data were required for inclusion. This meticulous selection process ensured a robust dataset for subsequent analyses 18.

### 2.2 Exclusion criteria

Exclusion criteria were established to maintain homogeneity within the study cohort. Patients with severe comorbid conditions, such as advanced pulmonary disease characterized by FEV₁/FVC < 0.5, were excluded, as were those with end-stage renal disease necessitating long-term dialysis, given the associated risks of metabolic derangements and fluid imbalance. Additionally, cases involving advanced hepatic failure (Child-Pugh grade C) were excluded due to significant impairments in coagulation, detoxification, and metabolic functions. Patients with preexisting advanced malignancies were also excluded, as their fragile physiological state, exacerbated by oncological therapies, could confound outcomes. Furthermore, those with unrelated severe congenital heart diseases, traumatic or iatrogenic aortic dissections, or significant intraoperative complications—such as uncontrollable hemorrhage or prolonged cardiac arrest—were excluded to ensure that the results specifically reflected the natural history and outcomes of ATAAD surgery 19, 20.

### 2.3 Grouping

To stratify patients based on postoperative complications, a retrospective review was conducted on 483 cases of acute lung injury (ALI) following surgical repair of ATAAD, performed at our institution between January 2020 and December 2023. Severe ALI was defined as an oxygenation index (OI) ≤ 100 mmHg within the first 72 hours postoperatively. The study population was divided into two groups: those who developed severe ALI (n = 182) and those who did not (n = 301). This stratification facilitated a focused investigation into risk factors associated with severe ALI, allowing for detailed comparisons between the two groups 21, 22.

### 2.4 Clinical information collection

Clinical data were comprehensively collected and included information on demographic characteristics, preoperative medical history, laboratory findings, and intraoperative parameters. Specific variables of interest included cardiopulmonary bypass (CPB) duration, moderate hypothermic circulatory arrest (MHCA) time, and aortic cross-clamp time, as well as biomarkers of inflammation and coagulation such as C-reactive protein (CRP), procalcitonin (PCT), D-dimer, and white blood cell (WBC) counts. This systematic data collection ensured a robust dataset for statistical analysis 23, 24.

### 2.5 Statistical methods

Statistical analyses were performed to identify meaningful associations and predictors of severe ALI. Normally distributed continuous variables were expressed as mean ± standard deviation and compared between groups using two-sample t-tests. To identify independent predictors of severe ALI, multivariate logistic regression analysis was employed, with results reported as odds ratios (ORs) and 95% confidence intervals (CIs). The diagnostic performance of these predictors was further evaluated using receiver operating characteristic (ROC) curve analysis, with the area under the curve (AUC) providing a measure of predictive accuracy^25^. A significance level of p < 0.05 was applied throughout the analysis to ensure robust statistical validity. These comprehensive methods were designed to provide a detailed understanding of the factors contributing to severe ALI in ATAAD patients, with the ultimate goal of informing strategies to improve clinical outcomes 26, 27.

## 3. Results

### 3.1 Comparisons of Clinical Characteristics Between Severe and Non-Severe ALI Groups

The comparison of clinical characteristics between patients with and without severe acute lung injury (ALI) revealed notable distinctions. No significant differences were observed in demographic variables such as age, gender, or in the prevalence of diabetes and hypertension between the severe ALI and non-severe ALI groups. However, the severe ALI group exhibited significantly higher proportions of patients with a preoperative oxygenation index (OI) ≤ 200 mmHg, smoking history, and coronary artery disease compared to their non-severe counterparts (P < 0.001, P = 0.032, and P = 0.039, respectively). In contrast, the prevalence of Marfan syndrome was notably lower in the severe ALI group (P = 0.033). Furthermore, elevated levels of body mass index (BMI), C-reactive protein (CRP), procalcitonin (PCT), D-dimer, and white blood cell count (WBC) were observed in the severe ALI group compared to the non-severe group (all P < 0.001, except PCT with P = 0.043). Additionally, key surgical parameters, including aortic cross-clamp duration, moderate hypothermic circulatory arrest (MHCA) time, cardiopulmonary bypass (CPB) duration, and length of ICU stay, were significantly longer in the severe ALI group (P < 0.01 for all comparisons) (Table 1).

### 3.2 Multivariate Logistic Regression Analysis of Risk Factors for Severe ALI

To identify predictors of severe ALI following ATAAD repair, a multivariate logistic regression model was constructed. Initially, univariate analyses were performed to evaluate the relationship between each variable and severe ALI. This preliminary analysis identified several potential risk factors, including preoperative OI, BMI, smoking history, coronary artery disease, Marfan syndrome, CRP, PCT, D-dimer, WBC count, aortic cross-clamp time, MHCA duration, and CPB time (Table 2). These candidate variables were subsequently analyzed using multivariate logistic regression to ascertain their independent contributions to severe ALI risk. The results demonstrated that preoperative oxygenation index was a significant independent predictor of severe ALI [P = 0.008, OR (95% CI): 0.002 (0.000-0.183)]. Similarly, BMI [P = 0.037, OR (95% CI): 1.569 (1.027-2.397)], CRP [P = 0.022, OR (95% CI): 1.292 (1.037-1.609)], D-dimer [P < 0.001, OR (95% CI): 3.841 (1.820-8.108)], MHCA time [P = 0.001, OR (95% CI): 3.306 (1.670-6.544)], and CPB duration [P = 0.017, OR (95% CI): 1.117 (1.020-1.223)] were identified as significant independent predictors (Table 3).

### 3.3 Diagnostic Accuracy of Predictive Variables for Severe ALI

**Figure 1:**
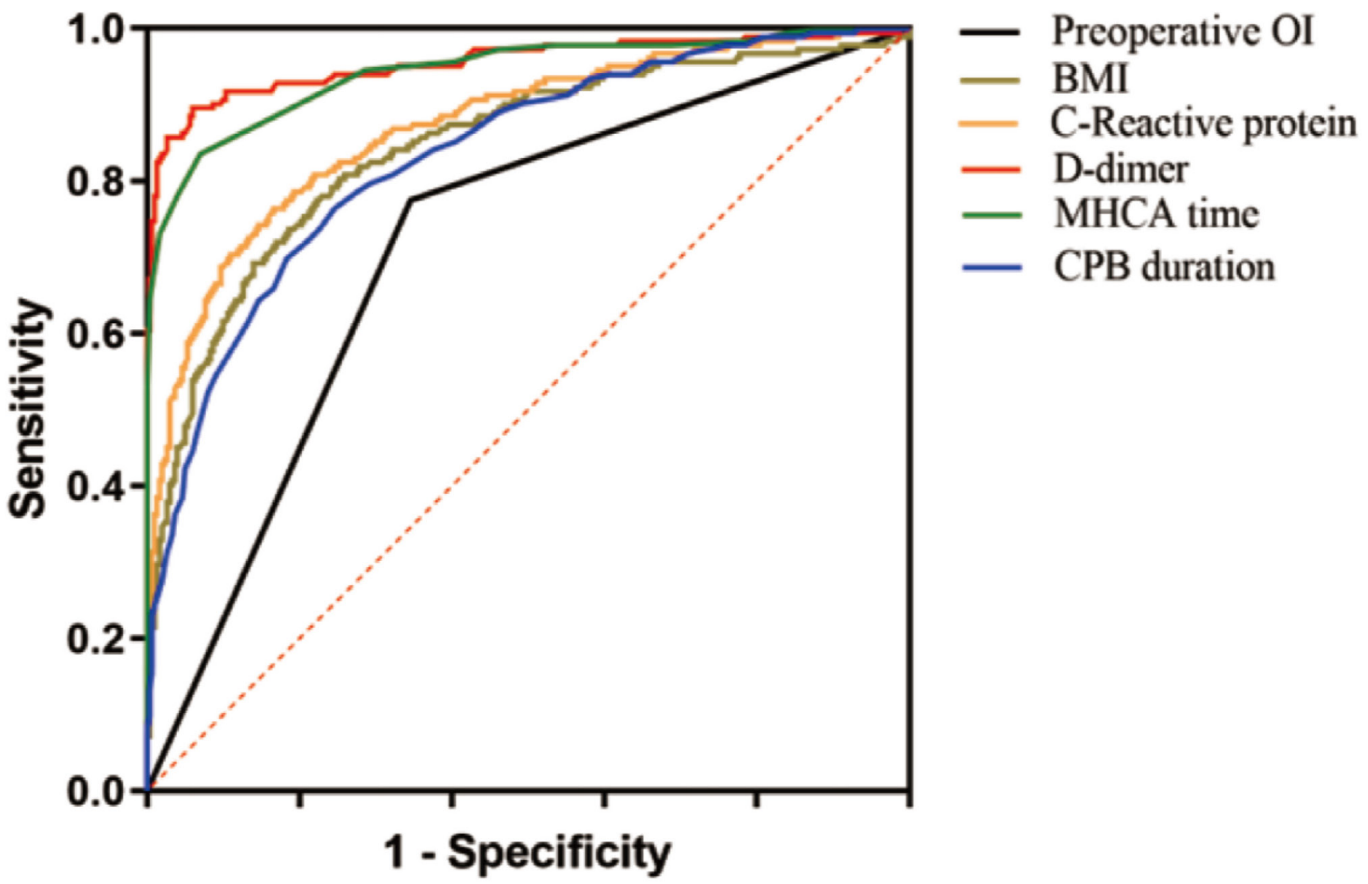
ROC analysis for severe ALI after ATAAD surgery.

The diagnostic performance of the predictive variables for severe ALI was evaluated through receiver operating characteristic (ROC) curve analysis. The area under the curve (AUC) values indicated that the predictive models based on preoperative oxygenation index, BMI, CRP, D-dimer, MHCA duration, and CPB time demonstrated excellent diagnostic capabilities, with AUC values of 0.715, 0.844, 0.871, 0.955, 0.944, and 0.833, respectively (all P < 0.001). These findings highlight the robustness of the identified predictors in assessing the likelihood of severe ALI following ATAAD surgery (Table 4 and Figure 1).

## 4. Discussion

The underlying mechanisms of acute lung injury (ALI) following acute type A aortic dissection (ATAAD) surgery are highly complex, with cardiopulmonary bypass (CPB) playing a pivotal role in its pathogenesis 28, 29. During CPB, a systemic inflammatory response is triggered, accompanied by complement system activation. This cascade produces anaphylatoxins that attract neutrophils to the lungs, where they release pro-inflammatory mediators such as reactive oxygen species, proteolytic enzymes, and cytokines 30, 31. These substances compromise the integrity of the alveolar-capillary membrane, leading to increased permeability and subsequent lung injury 32, 33. Additionally, ischemia-reperfusion injury (IRI) exacerbates this damage. The ischemic state of the lungs during CPB disrupts energy metabolism, and the subsequent reperfusion generates large amounts of oxygen free radicals, which attack cellular membranes and aggravate alveolar damage. Beyond the surgical process itself, postoperative factors also contribute to pulmonary damage 34, 35. For instance, a sharp rise in cardiac afterload during surgery increases left ventricular end-diastolic pressure and worsens pulmonary congestion 36, 37. Following aortic opening, hemodynamic fluctuations, hypotension, and reperfusion further aggravate the release of inflammatory mediators and microemboli, leading to pulmonary microvascular embolism and an inflammatory cascade 38.

Our findings identified preoperative oxygenation index (OI), body mass index (BMI), C-reactive protein (CRP), D-dimer levels, CPB duration, and moderate hypothermic circulatory arrest (MHCA) time as independent risk factors for ALI 39, 40. The mechanisms underlying these associations are multifaceted. Higher BMI reflects increased adipose tissue, which secretes inflammatory cytokines and mediators, predisposing obese patients to heightened inflammatory responses post-surgery 41, 42. This intensified inflammation damages vascular endothelial cells, increasing vascular permeability and promoting alveolar fluid leakage, which impairs gas exchange and elevates the risk of ALI 43, 44. Furthermore, poor thoracic compliance in obese patients limits respiratory function, compounding the difficulty of postoperative recovery and elevating ALI susceptibility 45, 46.

Elevated CRP levels, an acute-phase reactant, signify a robust inflammatory response. In the context of ATAAD, CRP >33.4 mg/L indicates systemic inflammation induced by surgical trauma, the implantation of artificial grafts, and postoperative stress responses 47. CRP activates the complement system, which recruits neutrophils to the lungs. The neutrophils release proteases and reactive oxygen species, damaging alveolar epithelial and pulmonary vascular endothelial cells 48. This increases alveolar-capillary permeability, leading to pulmonary edema and ALI. Similarly, elevated D-dimer levels (>10,109 ng/mL) reflect hyperactivation of the coagulation and fibrinolytic systems 49, 50. The dissection process itself exposes subendothelial collagen, triggering coagulation. Persistent hypercoagulable states promote microthrombus formation in pulmonary vasculature, causing localized ischemia and hypoxia 51-53. Concurrently, fibrinolytic system activation produces degradation products that damage lung tissue and perpetuate inflammation, further increasing vascular permeability and exacerbating ALI 54-57.

Preoperative OI serves as a critical indicator of baseline pulmonary function, and poor preoperative lung function, potentially due to underlying chronic pulmonary diseases or cardiac insufficiency, increases the risk of postoperative ALI. Reduced lung reserve impairs the ability to tolerate surgical trauma, CPB, and other intraoperative stressors, making the lungs more vulnerable to alveolar collapse, pulmonary edema, and subsequent ALI. Additional perioperative factors, such as anesthetic drugs and CPB-induced hemodilution, may further compromise lung function 58-61.

Prolonged CPB time (>5.69 hours) significantly increases the risk of ALI due to prolonged exposure of blood to artificial surfaces, which activates leukocytes and platelets, triggering the release of inflammatory cytokines like interleukin-1 and interleukin-6. These inflammatory mediators damage pulmonary vascular endothelial cells, increasing vascular permeability 62. Moreover, extended CPB time exacerbates ischemia-reperfusion injury, as ischemic lung tissue generates oxygen free radicals during reperfusion, which attack the alveolar and vascular endothelial cells, causing structural damage 63. Similarly, extended MHCA time disrupts pulmonary vascular regulation, increasing vascular permeability and promoting the leakage of fluid into the alveoli and interstitium, thereby impairing gas exchange and heightening ALI risk 64. In conclusion, the interplay between preoperative pulmonary function, systemic inflammation, coagulation abnormalities, and intraoperative factors such as CPB and MHCA significantly contributes to the development of ALI following ATAAD surgery. These findings underscore the importance of optimizing preoperative and perioperative strategies to mitigate risk factors and improve postoperative outcomes.

Despite the significant findings, this study has several limitations that must be acknowledged. First, the retrospective nature of the analysis may introduce inherent biases in data collection and interpretation. Although the inclusion and exclusion criteria were strictly defined, unmeasured confounding factors might still influence the results. Second, the study was conducted at a single institution, which may limit the generalizability of the findings to other populations and healthcare settings. Moreover, some variables, such as preoperative pulmonary function and inflammatory markers, were not dynamically monitored, which could have provided additional insights into the progression of acute lung injury (ALI). Lastly, while we identified independent risk factors for ALI, causality cannot be definitively established due to the observational design of the study.

Building on the findings of this study, several avenues for future research are evident. Prospective, multicenter studies with larger sample sizes are warranted to validate the identified risk factors and enhance the generalizability of the results. Furthermore, the inclusion of serial measurements of biomarkers, such as inflammatory cytokines and oxidative stress markers, could provide a more comprehensive understanding of the pathophysiological mechanisms underlying ALI. Investigating the role of preoperative interventions, such as optimized respiratory therapy or targeted anti-inflammatory treatments, may also yield strategies to mitigate ALI risk. Additionally, advanced imaging techniques and machine learning models could be employed to develop predictive algorithms, enabling early identification of high-risk patients and facilitating personalized perioperative management.

This study offers a novel contribution to the understanding of ALI in the context of acute type A aortic dissection (ATAAD) surgery. By systematically identifying preoperative oxygenation index, body mass index, C-reactive protein, D-dimer levels, CPB duration, and MHCA time as independent predictors of ALI, this research provides actionable insights that could inform perioperative decision-making. The use of receiver operating characteristic (ROC) curve analysis further underscores the predictive value of these factors, offering a practical framework for risk stratification. Importantly, the study highlights the interplay between inflammatory responses, coagulation abnormalities, and mechanical factors in ALI pathogenesis, advancing the current understanding of this multifaceted complication. These findings not only contribute to the academic literature but also hold significant clinical implications for improving patient outcomes in ATAAD surgery.

This study identified preoperative oxygenation index (OI), body mass index (BMI), C-reactive protein (CRP), D-dimer levels, moderate hypothermic circulatory arrest (MHCA) time, and cardiopulmonary bypass (CPB) duration as independent risk factors for severe acute lung injury (ALI) following ATAAD surgery. Specifically, a preoperative OI ≤ 200 mmHg, elevated BMI, increased CRP levels, and high D-dimer concentrations were associated with a significantly elevated risk of developing severe ALI. Conversely, shorter MHCA and CPB durations were found to mitigate the likelihood of severe ALI. These findings highlight the potential diagnostic and therapeutic value of these risk factors in predicting and managing severe ALI in patients undergoing ATAAD surgery, offering valuable insights for improving patient outcomes through targeted perioperative strategies.

## Figures and Tables

**Table 1:** Differences in clinical data between the severe ALI and non-severe ALI groups

Metric	Severe ALI group (n=182)	Non-severe ALI group (n=301)	t/χ^2^	P
Age (years)	45.96 ± 4.63	46.17 ± 4.55	0.488	0.626
Gender, n (%)			0.256	0.613
Male	90 (49.45%)	156 (51.83%)		
Female	92 (50.55%)	145 (48.17%)		
Preoperative OI, n (%)			83.595	<0.001
≤200 mmHg	141 (77.5%)	104 (33.9%)		
>200 mmHg	41 (22.5%)	197 (66.1%)		
BMI, kg/m^2^	26.43 ± 3.49	21.93 ± 2.59	16.186	<0.001
Smoking history, n (%)			4.619	0.032
Yes	82 (45.1%)	106 (35.2%)		
No	100 (54.9%)	195 (64.8%)		
Coronary heart disease, n (%)			4.267	0.039
Yes	32 (17.6%)	33 (11.0%)		
No	150 (82.4%)	268 (89.0%)		
Diabetes, n (%)			0.325	0.596
Yes	50 (27.47%)	90 (29.90%)		
No	132 (72.53%)	211 (70.10%)		
Hypertension, n (%)			0.006	0.936
Yes	35 (19.2%)	57 (18.9%)		
No	147 (80.8%)	244 (81.1%)		
Marfan syndrome, n (%)			4.571	0.033
Yes	3 (1.6%)	17 (5.6%)		
No	179 (98.4%)	284 (94.4%)		
C-Reactive protein (mg/L)	37.64 ± 7.95	26.31 ± 6.12	17.574	<0.001
PCT (ng/L)	10.36 ± 2.26	9.86 ± 2.82	2.030	0.043
D-dimer (mg/L)	12.47 ± 2.59	7.62 ±1.21	27.859	<0.001
WBC (10^9^/L)	12.98 ± 2.75	10.87 ± 2.63	8.398	<0.001
Aortic cross clamp duration (min)	85.52 ± 7.60	83.18 ± 8.14	3.138	0.002
MHCA time (min)	33 (30, 35)	23 (21, 26)	16.371	<0.001
CPB duration (min)	200.62 ± 14.76	181.15 ± 13.70	14.697	<0.001
Length of ICU (h)	216.61 ± 24.33	96.76 ± 18.35	61.356	<0.001
Length of hospitalization (day)	19 (17, 20)	18 (15, 20)	1.917	0.055

ALI: acute lung injury; OI: oxygenation index; BMI: Body mass index; PCT: Procalcitonin; WBC: White blood cell count; CPB: cardiopulmonary bypass; MHCA: moderate hypothermic circulatory arrest

**Table 2:** Univariate logistic regression analysis

Factor	Univariate logistic regression analysis				
	B	S.E.	Wald	P	OR (95%CI)
Preoperative OI	1.874	0.215	76.058	<0.001	6.514 (4.275-9.926)
BMI	0.444	0.041	118.556	<0.001	1.559 (1.439-1.689)
Smoking history	0.411	0.192	4.598	0.032	0.663 (0.455-0.965)
Coronary heart disease	0.550	0.268	4.198	0.040	0.577 (0.341-0.976)
Marfan syndrome	1.273	0.633	4.039	0.044	3.572 (1.032-12.361)
C-Reactive protein	0.233	0.021	118.158	<0.001	1.262 (1.210-1.316)
PCT	0.073	0.036	4.071	0.044	1.076 (1.002-1.155)
D-dimer	1.401	0.138	103.798	<0.001	4.060 (3.101-5.316)
WBC	0.294	0.040	54.162	<0.001	1.342 (1.241-1.452)
Aortic cross clamp duration	0.037	0.012	9.495	0.002	1.038 (1.014-1.063)
MHCA time	0.564	0.051	121.297	<0.001	1.758 (1.590-1.943)
CPB duration	0.098	0.009	106.584	<0.001	1.103 (1.083-1.124)
Length of ICU	1.020	1.189	0.736	0.391	2.774 (0.270-28.531)

B: Beta coefficient; S.E.: Standard Error; OR: Odds Ratio; CI: Confidence Interval

**Table 3:** Multivariate logistic regression analysis

Factor	Multivariate logistic regression analysis				
	B	S.E.	Wald	P	OR (95%CI)
Preoperative OI	6.484	2.443	7.043	0.008	0.002 (0.000-0.183)
BMI	0.450	0.216	4.332	0.037	1.569 (1.027-2.397)
Smoking history	0.432	1.176	0.135	0.713	0.649 (0.065-6.510)
Coronary heart disease	0.596	2.809	0.045	0.832	0.551 (0.002-135.452)
Marfan syndrome	1.579	5.445	0.084	0.772	4.852 (0.00-8887.211)
C-Reactive protein	0.256	0.112	5.213	0.022	1.292 (1.037-1.609)
PCT	0.009	0.245	0.001	0.971	1.009 (0.624-1.630)
D-dimer	1.346	0.381	12.468	<0.001	3.841 (1.820-8.108)
WBC	0.188	0.261	0.521	0.471	1.207 (0.724-2.011)
Aortic cross clamp duration	0.186	0.101	3.436	0.064	1.205 (0.989-1.467)
MHCA time	1.196	0.348	11.783	0.001	3.306 (1.670-6.544)
CPB duration	0.110	0.046	5.662	0.017	1.117 (1.020-1.223)

B: Beta coefficient; S.E.: Standard Error; OR: Odds Ratio; CI: Confidence Interval

**Table 4:** ROC analysis for severe ALI after ATAAD surgery

Variable	AUC	P value	95% CI
Preoperative OI	0.715	<0.001	0.667-0.762
BMI	0.844	<0.001	0.806-0.882
C-Reactive protein	0.871	<0.001	0.837-0.905
D-dimer	0.955	<0.001	0.933-0.977
MHCA time	0.944	<0.001	0.921-0.966
CPB duration	0.833	<0.001	0.796-0.870

AUC: Area under the curve; CI: Confidence Interval
